# 6-Gingerol Regulates Hepatic Cholesterol Metabolism by Up-regulation of LDLR and Cholesterol Efflux-Related Genes in HepG2 Cells

**DOI:** 10.3389/fphar.2018.00159

**Published:** 2018-02-27

**Authors:** Xiao Li, Jingting Guo, Ning Liang, Xinwei Jiang, Yuan Song, Shiyi Ou, Yunfeng Hu, Rui Jiao, Weibin Bai

**Affiliations:** Department of Food Science and Engineering, Institute of Food Safety and Nutrition, Guangdong Engineering Technology Center of Food Safety Molecular Rapid Detection, Jinan University, Guangzhou, China

**Keywords:** 6-gingerol, cholesterol metabolism, LDLR, cholesterol efflux, ABCA1

## Abstract

Gingerols, the pungent ingredients in ginger, are reported to possess a cholesterol-lowering activity. However, the underlying mechanism remains unclear. The present study was to investigate how 6-gingerol (6-GN), the most abundant gingerol in fresh ginger, regulates hepatic cholesterol metabolism. HepG2 cells were incubated with various concentrations of 6-GN ranging from 50 to 200 μM for 24 h. Results showed that both cellular total cholesterol and free cholesterol decreased in a dose-dependent manner. Besides, 6-GN ranging from 100 to 200 μM increased the LDLR protein and uptake of fluorescent-labeled LDL. Moreover, the mRNA and protein expressions of cholesterol metabolism-related genes were also examined. It was found that 6-GN regulated cholesterol metabolism via up-regulation of LDLR through activation of SREBP2 as well as up-regulation of cholesterol efflux-related genes LXRα and ABCA1.

## Introduction

Cardiovascular diseases (CVDs) are a major health hazard across the world, and high blood cholesterol level is closely related to CVD. The low-density lipoprotein (LDL) is a main carrier of blood cholesterol. It is well known that high level of low-density lipoprotein cholesterol (LDL-C) increases the risk of CVD, such as hyperlipidemia, atherosclerosis, and myocardial infarction (Ridker, 2014). Currently, statins are the most commonly used drugs to lower cholesterol levels via inhibition of HMG-CoA reductase (HMGCR) which is the rate-limiting enzyme for cholesterol *de novo* synthesis. In fact, many people on the brink of abnormal blood cholesterol level can return to normal blood cholesterol level without cholesterol-lowering medications. In recent years, the bioactive ingredients from functional foods have attracted much attention due to their potential as nutraceuticals to treat hypercholesterolemia (Chen et al., 2008).

Ginger serves as the homology of medicine and food for a long history in China and other Asian countries, and it has been reported to own a cholesterol-lowering activity in several animal studies (Verma et al., 2004; ElRokh et al., 2010; Nammi et al., 2010; Lei et al., 2014). Gingerols and shogaols, the pungent ingredients in ginger, are claimed to be the active compounds (Semwal et al., 2015; Zhang et al., 2017). 6-Gingerol (6-GN), which is the most abundant gingerol in fresh ginger (Lei et al., 2014), has been reported to have anti-cancerous, anti-oxidative, and anti-inflammatory bioactivities (Semwal et al., 2015; Amri and Touil-Boukoffa, 2016; Zhang et al., 2017).

Liver is an important organ for cholesterol metabolism (Maxwell et al., 2003). Numerous studies have proved that sterol regulatory element-binding proteins (SREBPs) and liver X receptors (LXRs) work together to maintain cholesterol homeostasis in the liver (Jiao et al., 2010). SREBP governs the transcription of HMGCR and low-density lipoprotein receptor (LDLR), with HMGCR acting as the rate-limiting enzyme in cholesterol *de novo* synthesis and LDLR being responsible for the removal of LDL-C from the circulation (Jiao et al., 2013). Moreover, proprotein convertase subtilisin/kexin type 9 (PCSK9), which can be also regulated by SREBPs, plays a major regulatory role in cholesterol homeostasis, mainly by reducing LDLR expression and preventing the uptake of cholesterol into the cells (Gustafsen et al., 2017). On the other hand, LXRs regulate the transcriptions of cholesterol-7α hydroxylase (CYP7A1) and ATP-binding cassette transporter ABCA1 (Chen et al., 2008; Jiao et al., 2010). CYP7A1 is known to convert cholesterol to bile acids for elimination, and ABCA1 transfers cholesterol from macrophages to lipid-poor apolipoproteins A-1 (apoA-1 and apoE), which then form nascent high-density lipoproteins (HDLs) (Sparrow et al., 2002; Zhang et al., 2002; Chen et al., 2008). However, to date, little is known about the effect of 6-GN on cholesterol metabolism in the liver.

The human HepG2 cell line was often used as a model to investigate the hepatic cholesterol metabolism. The present study was therefore to investigate the underlying mechanism of cholesterol-lowering effect of 6-GN and focus on the interaction of 6-GN with the mRNA and protein expressions of SREBP2, HMGCR, LDLR, PCSK9, LXRα, CYP7A1, and ABCA1 in HepG2 cells.

## Materials and Methods

### Cell Culture and Treatments

The human hepatocellular carcinoma cell line, HepG2, was obtained from the American Type Culture Collection (ATCC; Manassas, VA, United States) and cultured in DMEM (Gibco, Rockville, MD, United States) supplemented with 10% (v/v) fetal bovine serum (FBS; Gibco, Lofer, Austria), 1% of 100 U/ml penicillin/100 μg/ml streptomycin solution. The cells were incubated at 37°C under humidified 5% CO_2_. 6-GN (purity > 99%) was purchased from Chem-strong Company (Shenzhen, China). For each experiment, the concentrations of 6-GN were diluted with culture medium from the stock solution, which was dissolved in absolute alcohol, to the maximum final concentration of absolute alcohol at 0.1%.

### Cell Viability Analysis

The cytotoxic effect of 6-GN on HepG2 cells was evaluated by MTT assay. HepG2 cells were seeded in 96-well plates (1.5 × 10^4^ cells/well). After 12 h incubation, the cells were treated with or without various concentrations of 6-GN (50, 100, 200, 400, 600, and 800 μM) for 24 h, and incubated with 10 μl of MTT solution (5 mg/ml) for another 4 h. 150 μl of dimethyl sulfoxide was added instantly to each well and fully mixed for 10 min at room temperature. Then, the relative cell viability was quantified from the optical absorption read at 490 nm by using a microplate reader (Thermo Scientific, Chantilly, VA, United States).

### Cell Morphological Observation

For morphological observation, HepG2 cells were distributed in 6-well plates (1.5 × 10^5^ cells/well) for 12 h, then incubated with 0 (control), 100, and 200 μM 6-GN for 24 h. Cell shape was observed with a fluorescence microscope (Olympus, ×71).

### Cholesterol Determination in Cells

HepG2 cells were equally seeded in 6-well plates at a density of 6 × 10^5^. Then, the adherent cells were treated with 0 (control), 50, 100, 150, and 200 μM of 6-GN for 24 h. To measure total and free cholesterol in cells, Cholesterol Quantitation Kit (Sigma) was used. First, 200 μl of chloroform:isopropanol:IGEPAL CA-630 (7:11:0.1) was added to extract the cholesterol in cells. Then, the samples were centrifuged at 13,000 × *g* for 10 min to remove insoluble material, and the organic phase was volatilized under N_2_ at 50°C. Next, dried lipid samples were dissolved with 200 μl of the Cholesterol Assay Buffer, and vortex until the mixture was homogenous. At last, the samples and cholesterol standards were all detected with the function of Reaction Mixes by microplate reader according to the manufacturer’s protocol. The assays will detect total cholesterol (cholesteryl esters plus free cholesterol) in the presence of cholesterol esterase or only free cholesterol in the absence of the esterase enzyme.

### LDL Uptake Assay

HepG2 cells were cultured in 96-well plates with 1.5 × 10^4^ cells/well. Then, the cells were treated with 0 (control), 100, and 200 μM of 6-GN for 24 h, and the culture media was removed and replaced with 100 μl/well LDL-DyLight^TM^ 550 (Cayman) working solution diluted 1:1000 in serum-free culture medium. After incubation for 6 h at 37°C, the culture medium was removed and replaced with fresh culture medium. The degree of LDL uptake was examined under a microscope (Olympus) with filters capable of measuring excitation and emission wavelength 540 and 570 nm, respectively. Subsequently, the cells were ready for the immunocytochemical staining of LDL receptors. After 10 min incubation with 100 μl/well of cell-based assay fixative solution, the cells were washed by TBST three times for 5 min each. Next, the cells were incubated with 100 μl/well of cell-based assay blocking solution for 30 min, and with 100 μl/well of diluted Rabbit Anti-LDL Receptor Primary Antibody for another 1 h. After washed with TBST three times for 5 min each, the cells were incubated in the dark for 1 h with 100 μl/well of diluted DyLight^TM^ 488-Conjugated Secondary Antibody. At last, the cells were washed with TBST three times for 5 min each, and examined using a fluorescence microscope with a filter capable of excitation and emission at 485 and 535 nm, respectively.

### Real-Time PCR Analysis

Total RNA was isolated from HepG2 cells by a Hipura Total RNA Mini Kit (Magen), as recommended by the manufacturer’s instructions. Then, the complementary DNA (cDNA) was synthesized from total RNA with a PrimeScript^TM^ RT Reagent Kit (TaKaRa) and Real-Time PCR analysis was conducted with SYBR Green fluorescent dye method. Amplification cycles were 95°C for 30 s, 95°C for 5 s, and 60°C for 60 s. Primers for human SREBP2, HMGCR, LDLR, PCSK9, LXRα, CYP7A1, ABCA1, and β-actin were designed on the basis of GenBank database with the Primer Express software. The sequences of primer were listed in **Table 1**. The relative mRNA levels were obtained after normalizing to the β-actin expression.

**Table 1:** The primer sequences of related genes.

Gene	Forward primer (5’–3’)	Reverse primer (5’–3’)
SREBP2	AAGTCTGGCGTTCTGAGGAA	AGGTCCACCTCATTGTCCAC
HMGCR	CTTGTGTGTCCTTGGTATTAGAGCTT	GCTGAGCTGCCAAATTGGA
LDLR	CAAAGTCTGCAACATGGCTAGAGA	GTTGTCCAAGCATTCGTTGGTC
PCSK9	ATCCACGCTTCCTGCTGC	CACGGTCACCTGCTCCT
LXRα	GGAGGTACAACCCTGGGAGT	AGCAATGAGCAAGGCAAACT
CYP7A1	TTACAAGGCGGGACACACAG	CCTCAAGCTCTCTGCCAGTT
ABCA1	GACCCGCTGTATGGAAGGAA	CCAAGCATACGGGTTTGTGGA
β-Actin	TGGCACCCAGCACAATGAA	CTAAGTCATAGTCCGCCTAGAAGCA


### Western Blot Analysis

HepG2 cells were distributed in 6-well plates and treated with the indicated concentrations of 6-GN for 24 h. After incubation, cells were washed with PBS and lysed in ice cold radio immunoprecipitation assay lysis buffer (Cell Signaling, Beverly, MA, United States). Total proteins were extracted and measured according to the method described previously (Sun et al., 2016). Total proteins (20 μg per sample) were separated via 10% SDS–PAGE and transferred to polyvinylidene difluoride membranes. LDLR and PCSK9 were run together with β-actin in the same SDS–PAGE and transferred to the membrane; ABCA1, CYP7A1, and LXRα were run together with β-actin in the same SDS–PAGE and transferred to the membrane; SREBP2 and β-actin were run together in the same SDS–PAGE and transferred to the membrane; HMGCR and β-actin were run together in the same SDS–PAGE and transferred to the membrane. Then, the different protein bands in the same membrane were cut separately before binding the different primary antibodies. The membranes were then blocked in 1% nonfat milk (dissolved in Tris-buffered saline with Tween-20) for 1 h at room temperature, followed by incubation at 4°C overnight with the indicated primary antibodies: rabbit anti-human SREBP2 (1:1000; Abcam), goat anti-human LDLR (1:1000; R&D Systems), rabbit anti-human PCSK9 (1:2500; R&D Systems), rabbit anti-human HMGCR (1:1000; Absin), rabbit anti-human ABCA1 (1:1000; Absin), mouse anti-human LXRα (1:1000; R&D Systems), rabbit anti-human CYP7A1 (1:1000; Absin), and β-actin antibody (1:200; Santa). The expression of proteins was detected with corresponding species-specific HRP-labeled secondary antibodies (1:5000; Santa) and visualized by enhanced chemiluminescence (ECL; Thermo Scientific, Chantilly, VA, United States).

### Statistical Analysis

Values for all experiments were defined as the mean ± SEM. The significance of difference was evaluated by one-way ANOVA and *T*-test. Value of *p* < 0.05 (^∗^) was considered to be significant. All data were analyzed by GraphPad Software.

## Results

### Effect of 6-GN on Cell Viability in HepG2 Cells

**FIGURE 1:**
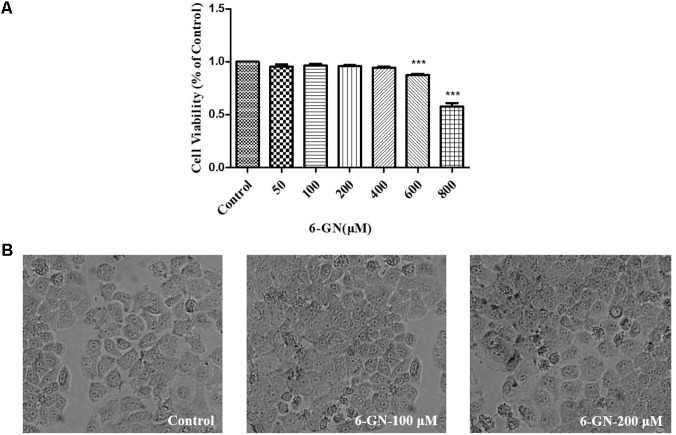
Effect of 6-GN on cell viability ratio and cell shape in HepG2 cells. **(A)** Cell viability ratio of HepG2 cells disposed with or without various concentrations of 6-GN (50, 100, 200, 400, 600, and 800 μM) for 24 h. **(B)** Cell shape disposed with 6-GN at the concentrations of 0 (control), 100, and 200 μM (200×). Values were represented as mean + SD of three independent experiments. ^∗∗∗^*p <* 0.001 versus control.

In order to estimate the effect of 6-GN on cell viability, HepG2 cells were incubated with 6-GN ranging from 0 to 800 μM for 24 h. 6-GN ranging from 50 to 400 μM had no effect on HepG2 cell viability, but 6-GN ranging from 600 to 800 μM significantly inhibited cell viability compared with control (**Figure 1A**). Also, as shown in **Figure 1B**, there was no significant effect on morphological changes at the concentration of 100 and 200 μM, which was consistent with the cell viability data. Therefore, 6-GN ranging from 0 to 200 μM was chosen as the proper concentrations for the following experiments.

### Effect of 6-GN on Total and Free Cholesterol Levels in HepG2 Cells

**FIGURE 2:**
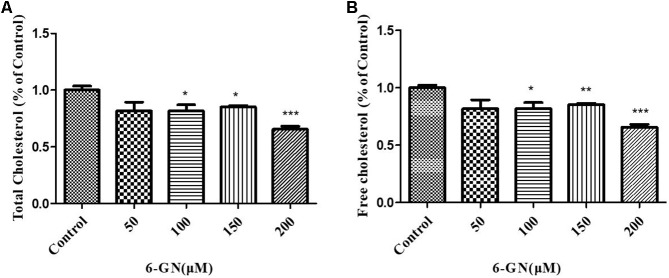
Effect of 6-GN on total and free cholesterol in HepG2 cells. **(A)** Total or **(B)** free cholesterol in HepG2 cells disposed with or without various concentrations of 6-GN (50, 100, 150, and 200 μM) for 24 h. Values were represented as mean + SD of three independent experiments. ^∗^*p* < 0.05 versus control, ^∗∗^*p* < 0.01 versus control, ^∗∗∗^*p* < 0.001 versus control.

HepG2 cells were incubated with various concentrations of 6-GN for 24 h. The concentrations of total cholesterol and free cholesterol in HepG2 cells are shown in **Figures 2A,B**. 6-GN decreased both total cholesterol and free cholesterol levels in a dose-dependent manner, significant differences were found at concentrations of 100, 150, and 200 μM.

### LDL Uptake Assay

**FIGURE 3:**
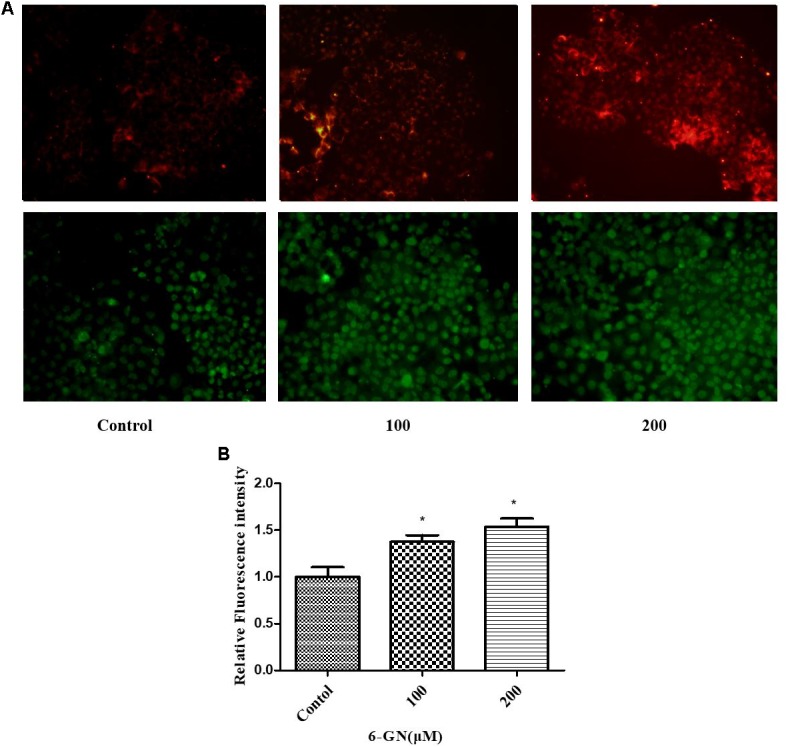
LDL uptake and LDLR-binding assay. **(A)** The degree of LDL uptake and LDLR fluorescence intensity in HepG2 cells disposed with or without various concentrations of 6-GN for 24 h (200×). **(B)** Relative fluorescence intensity of LDLR disposed with or without various concentrations of 6-GN. Values were represented as mean + SD of three independent experiments. ^∗^*p <* 0.05 versus control.

It was reported that LDLR must reach cell surface to bind with LDL-C and mediated its uptake (Spady, 1992). To investigate the underlying mechanisms involved in the cholesterol-lowering effect of 6-GN, the effects of 6-GN on LDLR activity in HepG2 cells were addressed. According to the results of cellular cholesterol determination assay, 6-GN at the effective concentrations of 100 and 200 μM was chosen for LDL uptake assay. As shown in **Figure 3A**, the degree of LDL uptake and LDLR-binding activity was both enhanced by 6-GN in a dose-dependent manner, significant differences were found in relative fluorescence intensity of LDLR between control and 6-GN-treated groups (**Figure 3B**).

### Results of Real-Time PCR Analysis

**FIGURE 4:**
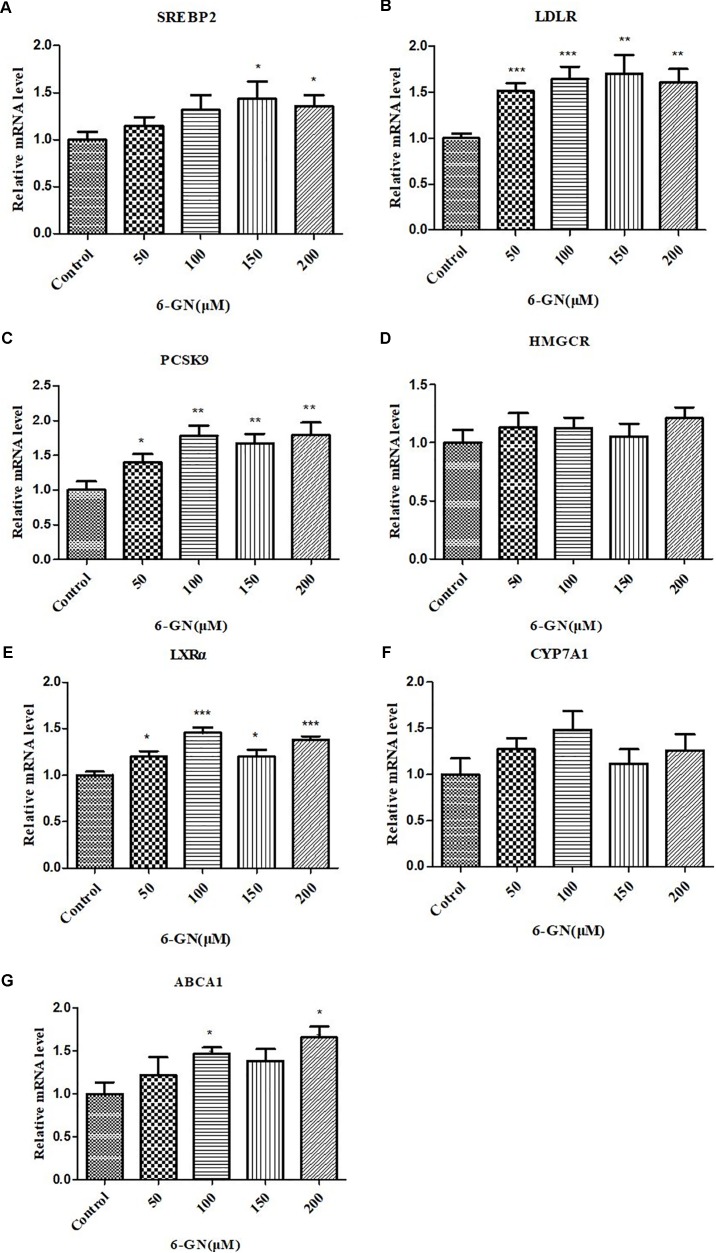
Effect of 6-GN on relative mRNA levels of **(A)** SREBP2, **(B)** LDLR, **(C)** PCSK9, **(D)** HMGCR, **(E)** LXRα, **(F)** CYP7A1, and **(G)** ABCA1 in HepG2 cells. HepG2 cells were disposed with or without various concentrations of 6-GN (50, 100, 150, and 200 μM) for 24 h. Values were represented as mean + SD of three independent experiments. ^∗^*p <* 0.05 versus control, ^∗∗^*p* < 0.0l versus control, ^∗∗∗^*p <* 0.001 versus control.

Real-time PCR was used to assess relative changes in the expression of selected genes related to cholesterol metabolism (**Figure 4**). 6-GN up-regulated the gene expression of SREBP2, LDLR, PCSK9, LXRα, and ABCA1, and had no significant effect on gene expression of HMGCR and CYP7A1 in HepG2 cells. The mRNA expressions of LDLR, PCSK9, and LXRα increased in all 6-GN-treated groups.

### Results of Western Blot Analysis

**FIGURE 5:**
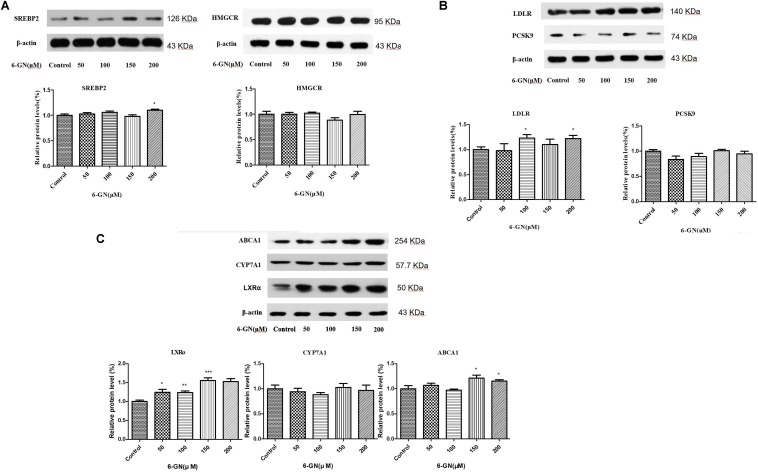
Effect of 6-GN on the related protein expressions of **(A)** SREBP2, HMGCR, **(B)** LDLR, PCSK9, **(C)** ABCA1, CYP7A1, and LXRα in HepG2 cells. HepG2 cells were disposed with or without various concentrations of 6-GN (50, 100, 150, and 200 μM) for 24 h (*n* = 3). Values were represented as mean ± SD of three independent experiments. *^∗^p <* 0.05 versus control, *^∗∗^p <* 0.01 versus control, ^∗∗∗^*p <* 0.001 versus control. LDLR and PCSK9 were run together with β-actin in the same SDS–PAGE and transferred to the membrane; ABCA1, CYP7A1, and LXRα were run together with β-actin in the same SDS–PAGE and transferred to the membrane; SREBP2 and β-actin were run together in the same SDS–PAGE and transferred to the membrane; HMGCR and β-actin were run together in the same SDS–PAGE and transferred to the membrane. Then, the different protein bands in the same membrane were cut separately before binding the different primary antibodies.

After 24 h incubation, 6-GN enhanced the protein levels of SREBP2, LDLR, LXRα, and ABCA1, and had no effect on PCSK9, HMGCR, and CYP7A1 (**Figure 5**). The protein levels of SREBP2 were up-regulated at the concentration of 200 μM and LDLR was up-regulated at the concentrations of 100 and 200 μM. However, no significant difference was found in protein expression of PCSK9, which was not consistent with its mRNA expression. The protein level of LXRα was up-regulated in a dose-dependent manner in 6-GN-treated groups. Moreover, 6-GN at the concentrations of 150 and 200 μM significantly enhanced the protein expression of ABCA1.

## Discussion

Elevated blood cholesterol level is a risk factor for CVD. Consequently, inhibition of cellular cholesterol transport, synthesis, and absorption, whether through drug (such as statins) or diet therapy, has been a primary treatment to reduce the risk of atherosclerosis. The present study examined the effect of 6-GN on cholesterol metabolism in HepG2 cells. The major observations in the present study were that 6-GN (ranging from 100 to 200 μM) could significantly reduce cellular total and free cholesterol levels, and also increase LDL uptake and LDLR-binding activity in HepG2 cells. The results regarding the cholesterol-lowering effect of gingerol were consistent with published data in several animal studies (Fuhrman et al., 2000; ElRokh et al., 2010; Elsm et al., 2010; Lei et al., 2014; Saravanan et al., 2014). The main finding of our present study was that cholesterol metabolism-related genes and proteins in the liver were modulated by 6-GN, which explains why 6-GN owns the cholesterol-lowering effect. The present study was the first to systematically investigate the underlying mechanisms by which 6-GN affected hepatic cholesterol metabolism.

Our study suggested that 6-GN regulated cholesterol metabolism via two pathways in the liver. The first pathway was that 6-GN up-regulated both gene and protein expressions of LDLR through activation of SREBP2. It is known that 70% of circulating LDL-C is removed by the liver, so hepatocytes are the major cells in LDLR-mediating uptake of circulating LDL-C (Goldstein et al., 2006). 6-GN could enhance cell capacity to uptake Dil-LDL and the LDLR-binding activity at the cell surface. It is well known that the gene expression of LDLR is governed by transcription factor SREBPs. When SREBP senses the cholesterol in endoplasmic reticulum (ER), it binds to two ER membrane proteins, insulin-inducing gene (INSIG) and SREBP cleavage-activating protein (SCAP) (Yang et al., 2002). Once the cholesterol levels fall, INSIG-1 dissociates from SREBP-SCAP, then SREBP-SCAP migrates to the Golgi apparatus and is cleaved by site-1 and -2 proteases. Subsequently, the resulted SREBP2 binds to the SRE in nucleus to turn on the transcription of LDLR (Goldstein et al., 2006; Hou et al., 2016). Therefore, in our study, 6-GN up-regulated both LDLR and SREBP2 at transcriptional and translational levels. HMGCR, the rate-limiting enzyme in cholesterol synthesis process, was also governed by SREBPs. However, no significant differences were found in both mRNA and protein levels of HMGCR in 6-GN-treated groups. So it is suggested that 6-GN might affect SCAP-SREBP2-LDLR pathway without changing cholesterol *de novo* synthesis. Several animal studies confirmed that ginger extract decreased serum LDL-C and hepatic cholesterol levels (ElRokh et al., 2010; Nammi et al., 2010; Lei et al., 2014). As we know that LDLR is responsible for the removal of LDL-C from the circulation to the liver for metabolism. Nammi et al. (2010) demonstrated that ethanol extract of *Zingiber officinale* (fresh ginger) up-regulated both hepatic LDLR mRNA and protein level, as well as down-regulating HMGR protein expression in rats. Our previous study found that dietary gingerol- and shogaol-enriched extract up-regulated the gene expression level of hepatic SREBP2 in hamsters; however, it had no effect on the mRNA and protein levels of LDLR and HMGCR (Lei et al., 2014). On the other hand, PCSK9 also regulates cholesterol metabolism via degradation of LDLR at the post-translational level. It is reported that statins can enhance the expression of PCSK9, which leads to the degradation of LDLR and thereby limiting the treatment effect of statins (Seidah et al., 2003; Dubuc et al., 2004). In our study, 6-GN up-regulated gene expression of PCSK9, but no effect on its protein level. A number of transcription factors or cofactors regulate the PCSK9 gene expression, including SREBP1/2 (Schulz et al., 2015). Therefore, up-regulation of PCSK9 gene expression may be related to activation of SREBP2 in 6-GN-treated groups.

The second pathway of 6-GN-mediated cholesterol metabolism was related to cholesterol efflux-related genes LXRα and ABCA1. ABCA1, an integral membrane protein, plays a key role in the regulation of cholesterol efflux (Smith et al., 2004; Yokoyama, 2006). Reverse cholesterol transport (RTC) is a multi-step process resulting in the movement of cholesterol from peripheral tissues back to the liver via the circulation (Li et al., 2013). Lipidation of apolipoprotein A-I (apoA-I) by ABCA1 is the rate-limiting step in RTC and generating plasma HDL (Aiello et al., 2003; Joyce et al., 2003). It is reported that the transcription of ABCA1 is regulated by LXRs and retinoid X receptor (RXR) (Costet et al., 2000; Schwartz et al., 2000). LXRα silencing reduced the response of LXR-target genes (such as ABCA1 and ABCG1) to LXR agonist and inhibited cholesterol efflux, but LXRβ had no such impact (Ishibashi et al., 2013). In the present study, 6-GN up-regulated both mRNA and protein levels of ABCA1 and LXRα in HepG2 cells. In contrast, our previous study found that gingerol and shogaol extract up-regulated hepatic CYP7A1, but down-regulated LXRα in hamsters, which was different with our present study (Lei et al., 2014). Although the present study didn’t show the same effect on CYP7A1 in HepG2 cells, gingerol indeed influenced cholesterol efflux-related genes in both animal and cell culture models.

In summary, we studied the effect of 6-GN on cholesterol metabolism in HepG2 cells. It was clear that 6-GN could decrease cellular total cholesterol and free cholesterol levels via up-regulation of LDLR through activation of SREBP2 as well as up-regulation of cholesterol efflux-related genes LXRα and ABCA1.

## Author Contributions

RJ was responsible for project design and the whole project and also wrote the manuscript. WB supported part of the experiment and also participated in the project design. SO supported part of the experiment and revised the manuscript. XL did the experiments and data analysis. JG did the Real-Time PCR part. NL designed the primer sequence for Real-Time PCR. XJ managed and conducted the work in our lab. YH conducted the work in our lab. YS conducted part of the experiment.

## Conflict of Interest Statement

The authors declare that the research was conducted in the absence of any commercial or financial relationships that could be construed as a potential conflict of interest.
